# Effect of Chinese Propolis as an Intracanal Medicament on Post-Operative Endodontic Pain: A Double-Blind Randomized Controlled Trial

**DOI:** 10.3390/ijerph17020445

**Published:** 2020-01-09

**Authors:** Juzer Shabbir, Fazal Qazi, Waqas Farooqui, Shahbaz Ahmed, Tazeen Zehra, Zohaib Khurshid

**Affiliations:** 1 Department of Operative Dentistry & Endodontics, Dow International Dental College, Dow University of Health Sciences, Karachi 74200, Pakistan; 2 Liaquat College of Medicine and Dentistry, Operative Dentistry Department, Karachi 75290, Pakistan; tazeenzehra@gmail.com; 3 Department of Operative Dentistry & Endodontics, Dr. Ishrat-ul-ibad Khan Institute of Oral Health Sciences, Dow University of Health Sciences, Karachi 72400, Pakistan; qazi.rehman@duhs.edu.pk (F.Q.); s.ahmed@duhs.edu.pk (S.A.); 4 School of Public Health, Dow University of Health Sciences, Karachi 74200, Pakistan; waqas.ahmed@duhs.edu.pk; 5 Department of Prosthodontics and Dental Implantology, College of Dentistry, King Faisal University, Al Ahsa 31982, Saudi Arabia

**Keywords:** calcium hydroxide, propolis, postoperative pain, natural product, clinical trial

## Abstract

Propolis is a potent anti-microbial and natural anti-inflammatory by-product obtained from the beehive. Studies have demonstrated the superior biocompatibility and anti-microbial properties of propolis as compared to calcium hydroxide. However, its effect on postoperative endodontic pain is unknown. Therefore, this study aimed to investigate the impact of Chinese propolis paste as an intracanal medicament on postoperative endodontic pain intensities compared with calcium hydroxide (control) at different time intervals in necrotic teeth with periapical radiolucency. Eighty patients with single-rooted necrotic teeth with visible periapical radiolucency were recruited and randomly allocated to either the calcium hydroxide or propolis groups. After chemo-mechanical preparation and intracanal medicament insertion, patients were given the VAS (visual analogue scale) to record pain scores. Inter-group data were compared and analyzed using two-way repeated measure ANOVA (Bonferroni test). A *p*-value of < 0.025 was considered significant. In total, >78% of the patients experienced no or only mild post-operative pain in both the groups at all time intervals, without any significant difference in pain scores between the two groups (*p* > 0.025). An overall flare-up rate of 14.8% was found. The results suggest that either of these medicaments can be used as an inter-appointment medication for the prevention of postoperative pain in necrotic cases.

## 1. Introduction

Pain is a common occurrence after endodontic treatment. Up to 40% of patients may experience some level of post-operative endodontic pain [1]. It is especially disturbing when the pain is initiated, or its intensity increases sharply after endodontic treatment. In the literature, such acute exacerbation of pain is coined a “flare-up” [2]. The rate of flare-up varies from 1.5% to 12.3% [3,4,5,6]. The etiology of postoperative pain is multifactorial and complex. It is related to iatrogenic and microbial factors. These factors cause irritation or injury to peri-radicular tissues. The extrusion of microbes and their byproducts from apical foramen during root canal preparation is the primary reason for the occurrence of severe post-instrumentation pain [7]. Therefore, to prevent such an undesirable situation, the operator should implement an effective anti-microbial strategy based on chemo-mechanical preparation along with the placement of inter-appointment intracanal medicaments, especially in infected cases.

Antimicrobial intracanal medicaments are considered as a method of disinfection of the root canal system [8]. In addition to antimicrobial effectiveness, the ideal intracanal medicament must possess an ability to prevent post-operative pain [9]. Calcium hydroxide is the most widely used anti-microbial intracanal medicament to which other medicaments are commonly compared to in the literature [10]. It has also been suggested that calcium hydroxide possess pain-relieving properties due to its antibacterial action and tissue-altering effects [11]. 

Recently, the focus of research has shifted towards finding natural alternatives to synthetic medications. Several natural products have been tested as intracanal medicaments for this purpose, namely: Liquorice extract, propolis, *Morinda controflia*, and the extract of *Arctium lappa* [12]. Propolis is a natural, biocompatible, and resinous byproduct, which has been used extensively as an organic medicine for managing oral and throat infection, dental caries, skin wounds, burns, leg ulcers, psoriasis, atopic dermatitis, recurrent aphthous ulcers, warts, herpes labialis, herpes genitalis, wound healing, tissue regeneration, and as a diet supplement [13]. Moreover, studies suggest that it can be used to prevent and treat the cases of oral mucositis induced due to cancer therapy [14] and to control diabetes and obesity complications [15]. It is prepared from substances collected from different parts of plants and deposited into a beehive by honey bees (*Apis mellifera* L.) [13]. It possesses anti-microbial and anti-inflammatory properties due to the presence of flavonoids. Moreover, the anti-microbial properties of propolis have been proven to be superior to calcium hydroxide in vitro [16,17]. Similarly, two clinical studies have found propolis to be superior to calcium hydroxide when used as a direct pulp capping agent and as an intracanal irrigant [18,19]. 

The mechanism by which propolis induces its anti-inflammatory and analgesic action is through the suppression of the lipopolysaccharide-induced inflammatory response of key cells [20]. Because of the potent anti-microbial and anti-inflammatory properties, propolis was expected to reduce the occurrence and intensity of inter-appointment pain. Therefore, the aim of the current trial was to compare the effect of propolis (paste) with calcium hydroxide paste on postoperative endodontic pain intensity in necrotic teeth with periapical pathosis at different time intervals. The null hypothesis was that there would be no difference in the pain severity between the two medicament groups. 

## 2. Materials and Methods

This study was a single-center, parallel-group, double-blind, randomized-controlled (1:1) clinical trial. It was structured and reported following the CONSORT statement. The study was approved by the Institutional Review Board (Ethics Committee) of Dow University of Health Sciences (ref no: IRB-847/DUHS/Approval/2017/52). The protocol of the study was registered at www.isrctn.com (Study ID: ISRCTN66816132) and the www.clinicaltrials.gov (Study Identifier: NCT03723980) database. The study was conducted from 16 November 2017 to 15 October 2018 in a secondary healthcare center, the out-patient department of operative dentistry/endodontics in Dow International Dental College, D.U.H.S, Karachi. All patients were informed about the trial in detail. Only those patients who signed the written informed consent form were included in the study. The primary outcome was defined as the mean pain score difference between the two groups at time intervals of 4, 12, 24, 48, and 72 h post-operatively. The secondary outcome was the evaluation of the incidence of a flare-up (an increase of ≥20 pain score points from one time interval to the next time interval [4]) according to time intervals in both groups. PASS v11 software (Microsoft, Redmond, WA, USA) was used to determine the sample size. For sample size estimation, pain scores of the calcium hydroxide group (2.42 ± 2.10) and creosote (1.12 ± 0.66) at 4 h was used from a previous study [21]. Two independent sample t tests were applied with 90% power of the test and a 95% confidence interval. The calculated minimum sample size was 52 (26 per group). To compensate for the expected drop out, the sample size was increased by 50%. Hence, the final sample size was 80 (40 in each group). The inclusion criteria consisted of: Patients aged 20 to 40 years having single-rooted necrotic teeth with symptomatic periapical periodontitis (pain score range = 1 to 100) and visible periapical widening or radiolucency. Exclusion criteria consisted of: Patients taking any medications that could have influenced pain perception, patients having painful conditions associated with more than one tooth, patients with the offending teeth having an unfavorable root morphology (open apex, severely curved, dilacerated, external or internal root resorption, severely sclerosed, and obliterated), patients with occlusal interferences related to the offending tooth, patients with medically compromised conditions (American Society of Anesthesiologists -III and above), patients with special communication needs, and patients allergic to bee pollen or honey products. A random sequence was generated online (www.random.org) using a simple randomization technique by an independent person. The allocation concealment was done by placing the sequence in a sealed envelope, which was then given to the dental assistant. The eligible patients were given serial numbers and assigned to the groups by the dental assistant according to the randomized sequence. The principal investigator, the patients, and the analyst (biostatistician) were blinded from the type of intracanal medicament used.

The teeth that did not respond to electric pulp testing (Electric pulp tester; Sybron endo, MI, USA) and cold sensibility test (Roeko ENDO-FROST cold spray; Coltene, Switzerland) were diagnosed as necrotic. The periapical diagnosis was made according to the *Glossary of Endodontic Terms* [2]. The healthy contralateral tooth was used as a control for sensibility and percussion tests. After confirming the eligibility, the affected tooth was anesthetized (with 2% lidocaine, 1.8 mL with 1:100,000 epinephrine. Medicaine Inj.) using the local infiltration technique (for maxillary teeth and mandibular anterior teeth) or inferior alveolar nerve block technique (for mandibular premolars), and isolated with a rubber dam. The dental caries was removed, and the pulp chamber was unroofed. The glide path was obtained with the help of an ISO stainless steel hand k-file #06, #08, and #10 number (Mani, Inc. Japan). The working length was taken with the help of an Apex locator (Dentaport ZX, J, Morita MFG CorpFushimi-ku, Kyoto, Japan) and confirmed with the periapical radiograph. The root canal preparation was carried out to #20 hand k-file (20/0.02 taper). Then, the Gates-Glidden burs were used to prepare the root canals coronally. The Rotary NiTi file system (Protaper universal; Dentsply Maillefer, Switzerland) was then used to prepare the root canals, with manufacturer’s recommended torque (3.5 N) and speed (300 rpm). The root canal preparation was carried out in small increments and minimum apical pressure with every effort to avoid over-instrumentation. Ethylene Diamine Tetra Acitic Acid (EDTA) (GlydeFile prep, Dentsply Maillefer, Switzerland) was used for lubrication during the procedure. The files were withdrawn and cleaned of debris intermittently. Recapitulation with the initial hand k-file and copious irrigation in a slow passive manner with 3% NaOcl (Canasol, Magnum Dental AS, Estonia) was carried out after each file was used. All the canals were prepared up to file size F1 (20/0.07). Then, apical gauging was carried out with an ISO NiTi k-file 20/0.02. If the file fit snugly at the apex, the preparation was terminated; if it was loose, the preparation was continued with the next larger rotary file and apical gauging was repeated with the corresponding hand k-file (0.02 taper). Using this method, 26% of the canals were prepared until F1, 55% till F2 (25/0.08), and 16% till F3 (30/0.09). Only 1% of the canals were prepared until F4 (40/0.06) and F5 (50/0.05). When this protocol was followed, all the canals were found to have a satisfactory apical stop. After completion of the root canal preparation, the canals were flooded with 17% EDTA solution (MD-Cleanser, Meta Biomed, Korea) for 3 min to remove the smear layer. Then, a final rinse with 5 mL of saline solution was done, and the canals were dried with sterile paper points (Dentsply Maillefer, Ballaigues, Switzerland). The principal investigator was responsible for the diagnosis, selection of patients, and root canal preparation. An experienced endodontist, acting as a secondary operator, was responsible for inserting the intracanal medicaments and temporary filling. Before the start of the study, he was given training for two weeks in mixing and inserting intracanal medicaments inside the plastic blocks (root canal simulator). 

**Figure 1:**
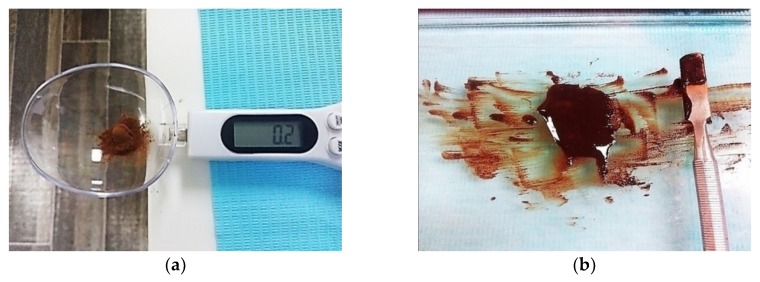
Propolis powder and propolis paste. (**a**) Weighing of propolis powder on a digital spoon scale. A two-scoop-level of (glass ionomer powder dispensing) the plastic spoon corresponded to 200 mg of propolis powder. (**b**) Propolis was mixed with saline to form a paste.

With the help of the randomized sequence, the dental assistant informed the secondary operator about the type of medicaments (characterized as group I and group II) to be inserted. The medicament group I consisted of teeth in which the usual standard of care, calcium-hydroxide paste (Calcipulpe, Septodont, Saint-Maur-des-Fossés, France), was inserted. It consisted of 20% calcium hydroxide, barium sulphate, and excipients. Approximately, 100 to 150 mg of calcium hydroxide were dispensed to be inserted in the root canal of a single tooth. The same paste has been used in previous studies as an intracanal medicament [22]. The medicament group II consisted of teeth in which the experimental/interventional intracanal medicament, propolis paste, was inserted. The propolis paste was formed by mixing 200 mg of 95% propolis powder (common name: Bee glue, brand name: FMBP. Henan Fumei Biotechnology Co., Ltd., Changge, China, reg no.: 411082100010933) with 0.3 mL of normal saline (1:1.5 wt./vol; Figure 1). A similar ratio of propolis and saline has been used by a previous study [23]. The type of propolis powder used in this study was “poplar type”. Flavonoids identified by the manufacturer in the product were: Quercetin, galanga, and chrysin. These medicaments were inserted into the root canals with the help of lentulo-spirals (Dentsply Maillefer, Switzerland). Excess medicaments were wiped off with damp cotton wool, making sure that no medicament remained above the Cementoenamel Junction Sterile dry cotton wool was then placed in the pulp chamber, and the cavity was filled with temporary cement (Cavit. ESPE Dental AG, Seefeld, Germany) by the secondary operator. 

Before dismissal, the patients were informed about, and given, the prescription of analgesics and the visual analogue scale (VAS). They were also educated regarding the adverse effects of codeine-containing medications and instructed to use these medications only if the OTC medicaments did not work. Patients self-recorded their pain intensity score on VAS pre-operatively, then at 4, 12, 24 (day 2), 48 (day 3), and 72 h (day 4). VAS consisted of a 100-mm line, with 0 indicating the absence of pain and 100 indicating the worst pain possible. The scale was further divided into 4 groups according to the severity of pain and requirement of analgesic: (1) 0 to 24 denoted as “no or only mild pain” with no requirement of analgesic; (2) 25 to 49 denoted as “moderate pain” requiring over-the-counter analgesic; (3) 50 to 74 denoted as “severe pain” requiring codeine-containing analgesic; and (4) 75 to 100 denoted as “extreme pain” with no medicine effective [4]. Patients were asked to record only the most intense pain experienced within the time period. For the guidance of patients, this criterion for the recording of pain was written on the VAS form. Additionally, the VAS scale did not contain identification of the medicament inserted, enforcing blinding of the principal investigator, who collected the VAS scales from the patients after 4 days. The VAS scales and the randomized sequence were given to the analyst (who was unaware about the type of medicaments registered for groups I and II) for analysis.

For data entry and analysis, IBM SPSS version 24 (IBM, Armonk, NY, USA) was used. For analysis of the distribution of age and pre-operative pain scores between the groups, the Mann–Whitney U test was applied; for the distribution of genders, the Chi-square test was applied; and for the distribution of the type of teeth, the Fisher exact test was applied. For multiple comparisons of the mean pain scores between groups and between time points, two-way repeated measure ANOVA with Bonferroni test was employed. A *p*-value < 0.025 was considered as significant. Descriptive statistics were reported for a flare-up and pain intensity according to each group.

## 3. Results

**Figure 2:**
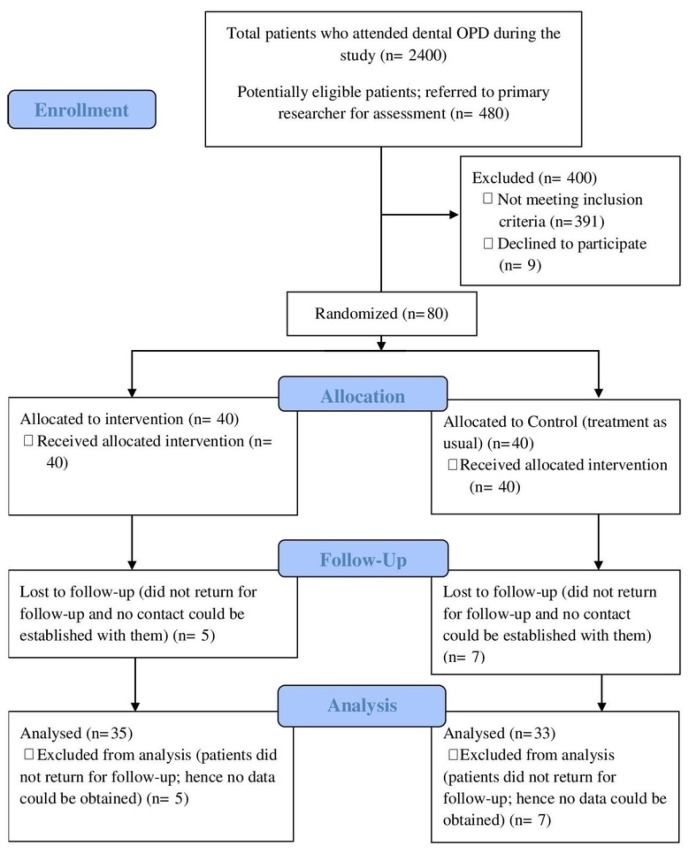
CONSORT patient flow diagram.

Analyses of the baseline characteristics revealed that both the study groups were homogenous in terms of age, gender, ethnicity, pre-operative pain status, and the type of teeth included in the study (*p* > 0.05, Table 1). There was an overall drop-out rate of 15% in the study. Therefore, the results of our study were based on 68/80 patients. The patient flow diagram is presented in Figure 2.

The difference in the mean pain scores between the two groups showed that patients in the propolis group had slightly more pain scores compared to the calcium hydroxide group at 12 h (−0.92), day 2 (−1.57), day 3 (−0.82), and day 4 (−0.72). Only at 4 h did the patients in the propolis group have lesser pain scores (2.96). However, the difference in pain scores between the groups was statistically non-significant at all the time intervals (*p* > 0.50) (Table 2). More than 78% of the patients in both groups experienced no or only mild pain (pain score = 0 to 24) throughout the follow-up period. Severe pain (pain score = 50 to 75) was experienced only by the patients in the calcium hydroxide group at 4 (9%) and 12 h (6%). In the control group, at 4 h, 12 % of the patients consumed OTC analgesics, and 9% consumed codeine-containing analgesics. At 12 h, 12% of the patients consumed OTC analgesics, and 6% consumed codeine-containing analgesics. At day 2 and 3, 6% and 3% of the patients consumed OTC analgesics, respectively. The remaining patients at these time intervals, and all the patients at day 4, did not consume any medication for pain relief. In the propolis group, at 4 h, 12 h, day 2, day 3, and day 4, 8.5%, 20%, 11%, 2.8%, and 5.7% of the patients consumed OTC analgesics. None of the patients in this group consumed codeine-containing analgesic, and the remaining patients did not consume any type of medication. To sum up, patients consumed oral analgesics only 9.6% of the times in the calcium hydroxide group and 9.7% of the time in the propolis group.

**Figure 3:**
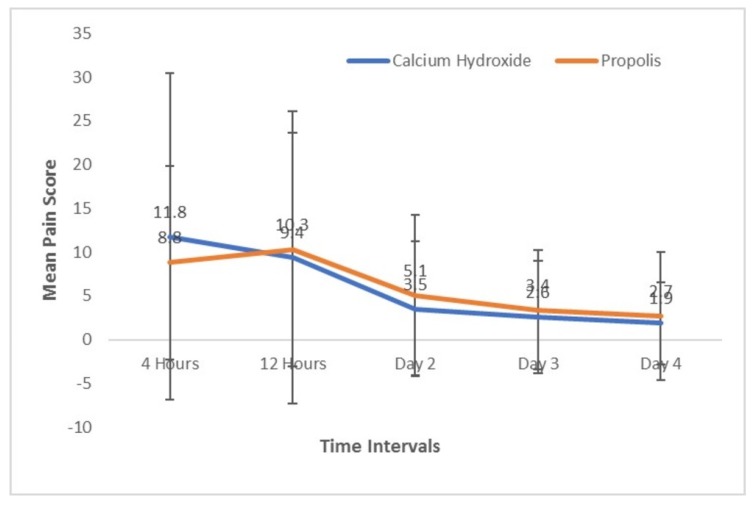
Pain score trend according to time.

The acute increase of pain scores (flare-up) was evaluated for four days. Overall, the incidence of flare-up was found to be 14.8% (Table 3), with a slightly higher flare-up rate in the propolis group (17%) compared to the calcium hydroxide group (12%). The mean pain scores were observed to be decreasing continuously with time. Surprisingly, in the propolis group, the pain scores of the patients were observed to increase slightly at the 12-h period (−1.5), only to decrease again from the next time interval (5.2). The mean pain scores in both groups were plotted against time and are presented in Figure 3. The study‘s dataset was made available to be viewed on Mendeley dataset page [24].

## 4. Discussion

In the literature, several methods have been described for the prevention of post-instrumentation pain. It includes the usage of the instrumentation technique that extrudes less debris, for instance, the crown-down technique with copious and frequent irrigation [25]; persistence of strict aseptic chain during all intracanal procedures [7]; anti-inflammatory drugs [26]; and intracanal medicaments [4,10,22]. Placing intracanal medicaments is one of the most conservative methods for reducing the microbial load and incidence of interappointment pain. In vitro studies regarding the anti-microbial [16,27,28], anti-inflammatory [29,30], and biocompatibility [31] properties along with other benefits of propolis due to its antioxidant effects have shown immense potential [32]. However, the effect of propolis on post-instrumentation pain was unknown. 

In the present study, necrotic teeth with periapical pathosis were specifically chosen for the study because intracanal medicaments are highly recommended in such teeth [33]. Single-rooted teeth were selected in this study to minimize iatrogenic errors as they are relatively easier to treat. Cases with symptomatic periapical periodontitis, with the presence of any level of pain on percussion (pain score = 1 to 100) were chosen because of its increased potential to cause postoperative pain [34]. The limited age range (20–40) was selected to limit the difference in pain perception due to differing ages [35]. The root canal preparation was carried out in a crown-down manner to limit the extrusion of debris and irrigant solution from the apex [25]. VAS was used to record pain because it is secure, reliable, and a frequently used subjective pain tool for the measurement of pain [4,36]. 

Overall, there was a continuous reduction of inter-appointment pain as time elapsed. However, the inter-group comparison revealed that pain intensities did not significantly differ between the two groups at any time interval. This is because most of the patients (>78%) experienced no or only mild inter-appointment pain (pain score <25) in both the medicament groups at all time intervals, which is in accordance with the results of a previous study. Additionally, the previous study also reported an insignificant difference in post-operative pain between the different intracanal medicament groups. However, due to the low incidence of inter-appointment pain, the authors recommended the use of either of those medicaments routinely [10]. The current result accepted the null hypothesis of the study.

The flare-up rate varies widely in the literature due to differences in the variables studied, and the methods used to characterize flare-up [3,4,6,37]. In the present study, a definition proposed by Ehrmann et al. was used to identify flare-up because of its well-defined criteria. The overall incidence of flare-up was found to be 14.8%, which was lower than the flare-up rate reported by Ehrmann et al. (19%) using the same definition. However, most of the flare-up cases in their study belonged to the no-treatment control group [4]. Although there was an increase of 20 or more points on the VAS scale, most of these patients fell only in the moderate pain intensity group (pain score <50). Nine out of 10 flare-up patients experienced moderate pain for which they consumed over-the-counter oral analgesic for pain relief. According to the definition proposed by the American Association of Endodontists, a flare-up is an acute exacerbation of pain [2]. The aim of the current definition of flare-up used in the study was to detect such acute exacerbation by giving it a numerical value. All the cases of flare-up, severe pain, and most cases of moderate pain quality occurred within 24 h post-operatively, which is in accordance with the literature [4,5,11,22]. This may be due to the slow action of these medicaments [23]. Interestingly, all the patients who experienced severe pain belonged to the calcium hydroxide group. However, their numbers were low.

One possible limitation of this study was the absence of spontaneous pain. The presence of pre-operative spontaneous pain is a predictive factor for postoperative pain [38]. However, in completely necrotic teeth, such pain is technically not possible. Another limitation of our study was the absence of the no-treatment group, which could have shown the difference between medicaments and no-medicament groups. 

## 5. Conclusions

Based on the findings, it can be concluded that there was no statistically significant difference in post-operative pain between calcium hydroxide and propolis groups. However, as the effect of propolis was observed to be similar to the calcium hydroxide group on postoperative endodontic pain without any adverse effects, it can be recommended for use as an intracanal medicament in necrotic cases. Additionally, in the future, a similar clinical trial is needed for a comparison of propolis with the no-treatment group, and other medicament groups.

## Figures and Tables

**Table 1:** Study sample characteristics.

Baseline Characteristics	Ca(OH)_2_ Group (n = 40)	Propolis Group (n = 40)	*p*-Value
Age	33.6 ± 6.3	33.2 ± 6	0.74
Gender			
*Males*	12 (30%)	18 (45%)	0.16
*Females*	28 (70%)	22 (55%)	
Pre-operative percussion pain	13.1 ± 17.4	18.1 ± 12.4	0.23
Type of Teeth			
*Maxillary Anterior*	16 (40%)	13 (32.5%)	0.21
*Maxillary 1st Premolar*	6 (15%)	14 (35%)	
*Mandibular Anterior*	4 (10%)	2 (5%)	
*Mandibular Premolar*	14 (35%)	11 (27.5%)	

**Table 2:** The difference in the mean pain scores between groups.

Time Interval	Ca(OH)_2_ Group Mean ± SD (Min–Max) (n = 33)	Propolis Group Mean ± SD (Min–Max) (n = 35)	Mean Difference (*p*-Value)
4 h	11.8 ± 18.7 (0–70)	8.8 ± 11.1 (0–43)	2.96 (>0.505)
12 h	9.4 ± 16.7 (0–50)	10.3 ± 13.3 (0–49)	−0.92 (>0.495)
Day 2	3.5 ± 7.7 (0–30)	5.1 ± 9.2 (0–30)	−1.57 (>0.495)
Day 3	2.6 ± 6.4 (0–30)	3.4 ± 6.8 (0–25)	−0.82 (>0.495)
Day 4	1.9 ± 4.7 (0–20)	2.7 ± 7.3 (0–30)	−0.72 (>0.495)

**Table 3:** Incidence of flare-up.

Time Interval	Ca(OH)_2_ Group (%) (n = 33)	Propolis Group (%) (n = 35)	Total (%)
4 h	4 (12%)	1 (2.8%)	5 (7.4%)
12 h	0	4 (11%)	4 (5.9%)
Day 2	0	1 (2.8%)	1 (1.5%)
Day 3	0	0	0
Day 4	0	0	0
Total	4 (12%)	6 (17%)	10 (14.8%)

## References

[1] Pak J.G., White S.N. (2011). Pain prevalence and severity before, during, and after root canal treatment: A systematic review. J. Endod..

[2] American Association of Endodontists (2016). Glossary of Endodontic Terms.

[3] Imura N., Zuolo M.L. (1995). Factors associated with endodontic flare-ups: A prospective study. Int. Endod. J..

[4] Ehrmann E.H., Messer H.H., Clark R.M. (2007). Flare-ups in endodontics and their relationship to various medicaments. Aust. Endod. J..

[5] Tsesis I., Faivishevsky V., Fuss Z., Zukerman O. (2008). Flare-ups after endodontic treatment: A meta-analysis of literature. J. Endod..

[6] Azim A.A., Azim K.A., Abbott P.V. (2017). Prevalence of inter-appointment endodontic flare-ups and host-related factors. Clin. Oral Investig..

[7] Siqueira J.F. (2003). Microbial causes of endodontic flare-ups. Int. Endod. J..

[8] Valverde M.E., Baca P., Ceballos L., Fuentes M.V., Ruiz-Linares M., Ferrer-Luque C.M. (2017). Antibacterial efficacy of several intracanal medicaments for endodontic therapy. Dent. Mater. J..

[9] Chong B.S., Pitt Ford T.R. (1992). The role of intracanal medication in root canal treatment. Int. Endod. J..

[10] Gama T.G.V., de Oliveira J.C.M., Abad E.C., Rôças I.N., Siqueira J.F. (2008). Postoperative pain following the use of two different intracanal medications. Clin. Oral Investig..

[11] Walton R.E., Holton I.F., Michelich R. (2003). Calcium hydroxide as an intracanal medication: Effect on posttreatment pain. J. Endod..

[12] Almadi E.M., Almohaimede A.A. (2018). Natural products in endodontics. Saudi Med. J..

[13] De Groot A.C. (2013). Propolis: A review of properties, applications, chemical composition, contact allergy, and other adverse effects. Dermatitis.

[14] Munstedt K., Mannle H. (2019). Using Bee Products for the Prevention and Treatment of Oral Mucositis Induced by Cancer Treatment. Molecules.

[15] Kitamura H. (2019). Effects of Propolis Extract and Propolis-Derived Compounds on Obesity and Diabetes: Knowledge from Cellular and Animal Models. Molecules.

[16] Shrivastava R., Rai V.K., Kumar A., Sinha S., Tripathi P., Gupta K., Sabharwal S. (2015). An in vitro Comparison of Endodontic Medicaments Propolis and Calcium Hydroxide alone and in Combination with Ciprofloxacin and Moxifloxacin against Enterococcus Faecalis. J. Contemp. Dent. Pract..

[17] Khurshid Z., Naseem M., Zafar M.S., Najeeb S., Zohaib S. (2017). Propolis: A natural biomaterial for dental and oral healthcare. J. Dent. Res. Dent. Clin. Dent. Prospect..

[18] Parolia A., Kundabala M., Rao N.N., Acharya S.R., Agrawal P., Mohan M., Thomas M. (2010). A comparative histological analysis of human pulp following direct pulp capping with Propolis, mineral trioxide aggregate and Dycal. Aust. Dent. J..

[19] Jolly M., Singh N., Rathore M., Tandon S., Banerjee M. (2013). Propolis and commonly used intracanal irrigants: Comparative evaluation of antimicrobial potential. J. Clin. Pediatr. Dent..

[20] Neiva K., Catalfamo D., Holliday S., Wallet S., Pileggi R. (2014). Propolis decreases lipopolysaccharideinduced inflammatory mediators in pulp cells and osteoclasts. Dent. Traumatol..

[21] Shah S.A., Maxood A., Shah S.I. (2010). Incidence of Endodontic Flare–UPS using either calcium hydroxide or Creosote as intracanal Medicament in Symptomatic teeth. J. Khyber Coll. Dent..

[22] Ehrmann E., Messer H., Adams G. (2003). The Relationship of Intracanal Medicaments to Postoperative Pain in Endodontics. Int. Endod. J..

[23] Chua E.G., Parolia A., Ahlawat P., Pau A., Amalraj F.D. (2014). Antifungal effectiveness of various intracanal medicaments against Candida albicans: An ex-vivo study. BMC Oral Health.

[24] Saifee J., Qazi F., Farooqui W., Ahmed S. (2019). Effect of Chinese Propolis as an Inter-Appointment Intracanal Medicament on Post-Instrumentation Pain: A Double Blind Randomized Controlled Trial.

[25] Aksel H., Kucukkaya Eren S., Cakar A., Serper A., Ozkuyumcu C., Azim A.A. (2017). Effect of Instrumentation Techniques and Preparation Taper on Apical Extrusion of Bacteria. J. Endod..

[26] Elzaki W.M., Abubakr N.H., Ziada H.M., Ibrahim Y.E. (2016). Double-blind Randomized Placebo-controlled Clinical Trial of Efficiency of Nonsteroidal Anti-inflammatory Drugs in the Control of Post-endodontic Pain. J. Endod..

[27] Xu X., Pu R., Li Y., Wu Z., Li C., Miao X., Yang W. (2019). Chemical Compositions of Propolis from China and the United States and their Antimicrobial Activities Against Penicillium notatum. Molecules.

[28] Rebia R.A., Binti Sadon N.S., Tanaka T. (2019). Natural Antibacterial Reagents (Centella, Propolis, and Hinokitiol) Loaded into Poly[(R)-3-hydroxybutyrate-co-(R)-3-hydroxyhexanoate] Composite Nanofibers for Biomedical Applications. Nanomater.

[29] Shahinozzaman M., Taira N., Ishii T., Halim M.A., Hossain M.A., Tawata S. (2018). Anti-Inflammatory, Anti-Diabetic, and Anti-Alzheimer’s Effects of Prenylated Flavonoids from Okinawa Propolis: An Investigation by Experimental and Computational Studies. Molecules.

[30] Governa P., Cusi M.G., Borgonetti V., Sforcin J.M., Terrosi C., Baini G., Miraldi E., Biagi M. (2019). Beyond the Biological Effect of a Chemically Characterized Poplar Propolis: Antibacterial and Antiviral Activity and Comparison with Flurbiprofen in Cytokines Release by LPS-Stimulated Human Mononuclear Cells. Biomedicines.

[31] Ramos I.F., Biz M.T., Paulino N., Scremin A., Della Bona Á., Barletta F.B., Figueiredo J.A.P. (2012). de Histopathological analysis of corticosteroid-antibiotic preparation and propolis paste formulation as intracanal medication after pulpectomy: An in vivo study. J. Appl. Oral Sci..

[32] Braakhuis A. (2019). Evidence on the Health Benefits of Supplemental Propolis. Nutrients.

[33] Law A., Messer H. (2004). An evidence-based analysis of the antibacterial effectiveness of intracanal medicaments. J. Endod..

[34] Hargreaves K.M., Hutter J. (2001). Endodontic pharmacology. Pathways of Pulp.

[35] Lautenbacher S., Peters J.H., Heesen M., Scheel J., Kunz M. (2017). Age changes in pain perception: A systematic-review and meta-analysis of age effects on pain and tolerance thresholds. Neurosci. Biobehav. Rev..

[36] Bourdel N., Alves J., Pickering G., Ramilo I., Roman H., Canis M. (2015). Systematic review of endometriosis pain assessment: How to choose a scale?. Hum. Reprod. Update.

[37] Relvas J.B., Bastos M.M., Marques A.A., Garrido A.D., Sponchiado E.C. (2016). Assessment of postoperative pain after reciprocating or rotary NiTi instrumentation of root canals: A randomized, controlled clinical trial. Clin. Oral Investig..

[38] Shresha R., Shrestha D., Kayastha R. (2018). Post-Operative Pain and Associated Factors in Patients Undergoing Single Visit Root Canal Treatment on Teeth with Vital Pulp. Kathmandu Univ. Med. J..

